# Transcription factor 4 expression in the developing non-human primate brain: a comparative analysis with the mouse brain

**DOI:** 10.3389/fnana.2024.1478689

**Published:** 2024-10-22

**Authors:** Alain C. Burette, Hanna Vihma, Audrey L. Smith, Siddhi S. Ozarkar, Jeff Bennett, David G. Amaral, Benjamin D. Philpot

**Affiliations:** ^1^Neuroscience Center, University of North Carolina at Chapel Hill, Chapel Hill, NC, United States; ^2^Department of Cell Biology and Physiology, University of North Carolina at Chapel Hill, Chapel Hill, NC, United States; ^3^Department of Psychiatry and Behavioral Sciences, MIND Institute, University of California, Davis, Davis, CA, United States; ^4^California National Primate Research Center, University of California, Davis, Davis, CA, United States; ^5^Carolina Institute for Developmental Disabilities, University of North Carolina at Chapel Hill, Chapel Hill, NC, United States

**Keywords:** primate, subgranular zone, Pitt-Hopkins syndrome, TCF4, schizophrenia

## Abstract

Transcription factor 4 (TCF4) has been implicated in a range of neuropsychiatric disorders, including major depressive disorder, bipolar disorder, and schizophrenia. Mutations or deletions in TCF4 cause Pitt-Hopkins syndrome (PTHS), a rare neurodevelopmental disorder. A detailed understanding of its spatial expression across the developing brain is necessary for comprehending TCF4 biology and, by extension, to develop effective treatments for TCF4-associated disorders. However, most current knowledge is derived from mouse models, which are invaluable for preclinical studies but may not fully capture the complexities of human neuropsychiatric phenotypes. This study compared TCF4 expression in the developing mouse brain to its regional and cellular expression patterns in normal prenatal, neonatal, and young adult rhesus macaque brains, a species more relevant to human neurodevelopment. While the general developmental expression of TCF4 is largely conserved between macaques and mice, we saw several interspecies differences. Most notably, a distinct layered pattern of TCF4 expression was clear in the developing macaque neocortex but largely absent in the mouse brain. High TCF4 expression was seen in the inner dentate gyrus of adult mice but not in macaques. Conversely, TCF4 expression was higher in the adult macaque striatum compared to the mouse striatum. Further research is needed to show the significance of these interspecies differences. Still, they underscore the importance of integrating rodent and primate studies to comprehensively understand TCF4 function and its implications for human disorders. Moreover, the primate-specific expression patterns of TCF4 will inform genetic and other therapeutic strategies to treat TCF4-associated disorders.

## 1 Introduction

Proper brain development relies on a complex genetic blueprint to ensure the precise orchestration of genes essential for brain formation and function through tightly controlled spatial and temporal expression of transcription factors. A crucial player in this process is Transcription Factor 4 (TCF4; OMIM 602272), also known as ITF2, SEF2, E2-2, but different from the canonical Wnt signaling-associated T-cell factor 4 encoded by the TCF7L2 gene. TCF4 is a member of the E-protein transcription factor family that binds to the regulatory DNA motif CANNTG. Its transcriptional activity relies on its interaction with various transcription factors, including proneural proteins that initiate neurogenesis in the early nervous system. For example, the proneural protein Math1 (also known as Atoh1) specifically depends on TCF4 for its role during brain development (Flora et al., 2007). TCF4 also forms inactive heterodimers with inhibitor of DNA binding 2 (previously known as inhibitor of differentiation 2), which prevents TCF4 from binding with its activators, effectively inhibiting its function (Langlands et al., 1997). The balance of E-proteins, proneural proteins, and inhibitors of differentiation proteins in a cell during development is crucial for determining future cell types (Powell and Jarman, 2008). Interestingly, TCF4 can also interact with non-bHLH transcription factors, as shown by its interaction with SOX11 (Moen et al., 2017; Wittmann et al., 2021).

Like other Type I bHLH proteins, TCF4 is expressed in various organs (Sepp et al., 2011). However, its expression is especially high in the brain, where it is present in most regions. Its expression is developmentally regulated, with peak levels during fetal development. In contrast with the other three E-proteins, its expression is maintained in the adult brain, albeit at much lower levels (Uittenbogaard and Chiaramello, 2000; Ravanpay and Olson, 2008; Brzozka et al., 2010; Li et al., 2018; Kim et al., 2020).

While TCF4 biology is still poorly understood, it is important for a wide array of functions. This versatility likely stems from its highly context-dependent interactions, which vary according to cell type, stage of development, and external signals. During brain development, TCF4 is important for neural stem cell differentiation (Fischer et al., 2014), and is implicated in the differentiation, maturation, and migration of neurons and subsequent synapse formation (Schmidt-Edelkraut et al., 2014; Li et al., 2019; Mesman et al., 2020; Schoof et al., 2020). TCF4 also plays an important role in the development of glial cells (Chen et al., 2021). It is required for proper survival and differentiation of oligodendrocytes and CNS myelination (Phan et al., 2020; Wedel et al., 2020). In the adult brain, TCF4 facilitates hippocampal adult neurogenesis (Shariq et al., 2021) and is needed in adult neurons to maintain normal structure and excitability (Sarkar et al., 2021). Additionally, TCF4 has been shown to regulate synaptic function and plasticity (Kennedy et al., 2016; Thaxton et al., 2018; Davis et al., 2023).

The importance of TCF4 is further underscored by its link to human disorders: deletions or specific mutations in the TCF4 gene cause Pitt-Hopkins syndrome (PTHS, OMIM #610954). Most mutations leading to PTHS are *de novo*, with occasional cases of parental mosaicism (Kousoulidou et al., 2013; Steinbusch et al., 2013), resulting in either reduced TCF4 function or dominant negative effects. Common characteristics of PTHS include intellectual disability, developmental delays, breathing issues, limited or no speech, motor delays, seizures, constipation, and distinct facial features (Whalen et al., 2012; Watkins et al., 2019). Additionally, individuals with PTHS often exhibit specific social behaviors and traits associated with autism spectrum disorder (Watkins et al., 2019). Besides their well-established role in PTHS, whole genome association studies show that TCF4 polymorphisms are also associated with schizophrenia (Schizophrenia Psychiatric Genome-Wide Association Study Consortium, 2011; Cross-Disorder Group of the Psychiatric Genomics Consortium, 2013; Kousoulidou et al., 2013), bipolar disorder (Del-Favero et al., 2002; Cross-Disorder Group of the Psychiatric Genomics Consortium, 2013), post-traumatic stress disorder (Gelernter et al., 2019), and major depression disorder (Cross-Disorder Group of the Psychiatric Genomics Consortium, 2013). Despite TCF4’s clinical importance, its biology is still largely unknown, and the precise mechanisms by which TCF4 mutations cause PTHS and other human disorders are still unclear.

Where TCF4 is located determines its interacting partners, its molecular function, and, hence, its cellular impact. Given this context-sensitive nature, understanding TCF4 biology and developing effective pharmacological or genetic approaches to treat TCF4-associated disorders require a detailed understanding of its spatial and temporal expression. Moreover, the absence of severe symptoms in mouse models carrying clinically relevant heterozygous TCF4 mutations, sometimes contrasting with the phenotypes seen in human patients (Thaxton et al., 2018), emphasizes the necessity of investigating potential interspecies differences in TCF4 expression between primates and rodents. This comparative analysis will be particularly crucial for developing therapeutic strategies aimed at addressing TCF4 haploinsufficiency in PTHS.

To advance translational research and obtain insights into TCF4 biology relevant to human neurodevelopment, we examined the spatiotemporal expression patterns of TCF4 in both developing and adult brains, comparing findings from mice and macaque monkeys, with the latter serving as a close proxy for human neurological development.

## 2 Methods

### 2.1 Mouse tissue

C57BL/6 J mice (18 female and 18 male) were deeply anesthetized with sodium pentobarbital (60 mg/kg, i.p.) and then intracardially perfused with phosphate-buffered saline (PBS, 0.1 M, pH 7.3). This was followed by a 10-min perfusion with 4% freshly depolymerized paraformaldehyde in phosphate buffer (pH 7.3). Brains were extracted, postfixed overnight at 4°C in the same fixative solution, cryoprotected in 30% sucrose in PBS, and sectioned at 50 μm using a sliding microtome.

### 2.2 Rhesus macaque tissue

Brain sections from eight macaque monkeys (*Macaca mulatta*) were used in this study at the following ages: gestational day (GD) 151, male; 14 days, female; 14 days, male; one month and three days, male; one month, male; two months and 24 days, male; three months and three days, male; 5 years four months and 14 days, male. The average pregnancy of a rhesus monkey is approximately 166 days. Sections were obtained from the tissue repository at the Amaral laboratory, and no animals were sacrificed specifically for this project. Tissue from these animals was used in several other studies (Jabes et al., 2010; Gonzalez Ramirez et al., 2024). The cryopreserved sections were stored at −80°C following the tissue preservation method of Rosene et al. (1986), which is optimized for long-term storage of valuable non-human primate tissue (Rosene et al., 1986). This method has been widely adopted by the Amaral laboratory and many other primate and human neuroanatomy laboratories for decades. Studies consistently show that tissue stored at −80°C experiences little to no degradation, with immunohistochemical staining performed after 10 or more years yielding virtually identical results. This preservation technique maximizes the use of valuable tissue and reduces the number of nonhuman primates needed for neuroscience research.

Briefly, animals were deeply anesthetized with sodium pentobarbital (50 mg/ kg i.v., Fatal-Plus, Vortech Pharmaceuticals, Dearborn, MI) and transcardially perfused. Postnatal cases underwent a “modified immuno-perfusion” involving sequential transcardial perfusion with phosphate buffered 1% paraformaldehyde at 4°C (250 mL/min for 2 min), followed by 4% paraformaldehyde at the same rate and temperature for 10 min, and concluding with 4% paraformaldehyde at 100 mL/min for 50 min. The brain was then extracted and postfixed in 4% paraformaldehyde for 6 h. Cryoprotection involved overnight immersion in 10% glycerol and 2% DMSO, followed by approximately 72 h in 20% glycerol with 2% DMSO. Finally, the brain was frozen in isopentane chilled with dry ice and ethanol. Fetal perfusions were adjusted with slower flow rates and shorter fixation durations (e.g., 75 mL/min for GD 151). Fetal brains were sectioned on a freezing sledge microtome without blocking, using cryogel for stage fixation and powdered dry ice for freezing. Other brains were blocked in the coronal plane at approximately −5 mm AP and affixed using OCT and phosphate buffer. A consistent sectioning scheme was used for all brains, cutting 30 μm sections in a 1:8 series. Series 1–3 and 5–8 were cryopreserved, while series 4 was fixed in 10% buffered formalin for 4 weeks before Nissl staining with thionin. Cryopreserved sections were stored at −80°C.

### 2.3 Western blotting

Tissues were snap-frozen in liquid nitrogen and stored at −80°C until lysis. Tissues were lysed using RIPA buffer (50 mM Tris–HCl pH 8.0, 150 mM NaCl, 1% NP-40, 0.5% Na-deoxycholate, 0.5% SDS) and protease inhibitor cocktail (P8340, Millipore Sigma) on ice. The samples were homogenized on ice using a Tissue Tearor (Model 985–370), and then centrifuged at maximum speed for 10 min at 4°C. Protein concentrations were measured using the BCA protein assay kit (Pierce). A total of 20 μg of each sample was separated in 4–20% Mini-PROTEAN TGX precast protein gel (Bio-Rad) by electrophoresis and transferred onto a 0.45 μm Immobilon-FL PVDF membrane (Millipore) in ice-cold transfer buffer (25 mM Tris-base, 192 mM glycine, and 20% MeOH) at 90 V for 90 min. Membranes were blocked in Intercept Blocking Buffer for 1 h and then blotted with primary antibodies: [anti-TCF4: ab130014 Abcam (Rabbit; 1:1000), ab217668 Abcam (Rabbit; 1:1000), sc-393407 Santa Cruz (Mouse; 1:1000), HPA025958 Millipore Sigma (Rabbit; 1:1000), SAB1412620 Millipore Sigma (Mouse;1:1000), LS-C331289 LS Bio (Rabbit; 1:1000), LS-B8267 LS Bio (Rabbit; 1:1000), C48H11 Cell Signaling (Rabbit; 1:1000), and anti-β-Tubulin (1:10,000, ab6046, Abcam)] on a shaker overnight at 4°C. The next day, the membranes were washed with PBS/0.5% Tween-20 three times and incubated with anti-mouse or anti-rabbit HRP-conjugated secondary antibodies (1:5000, Invitrogen anti-mouse 31430 or anti-rabbit 31460) for 1 h at RT, and then washed with PBS/0.5% Tween-20 three times. Chemiluminescence reaction was performed using Clarity Western ECL Substrate (Bio-Rad), or Super Signal West Atto chemiluminescent substrate (Thermo A38554), which was imaged by an Amersham Imager 680 (GE Healthcare).

### 2.4 Short hairpin RNA knockdown experiments

**Figure 1:**
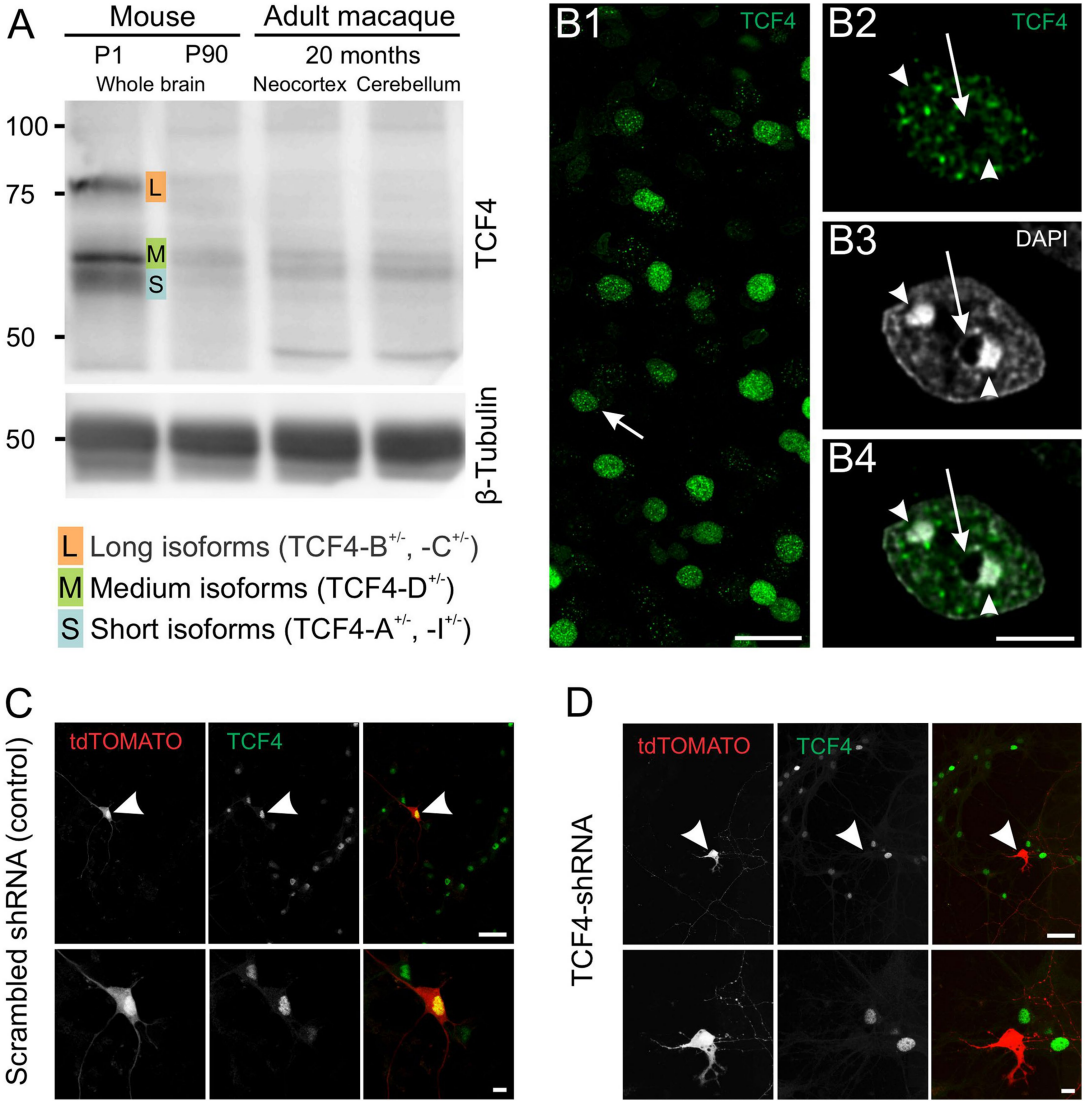
Validation of TCF4 antibody NCI-R159-6 (ab217668). **A**: Western blot analysis demonstrating the specificity of ab217668 for long (L), medium (M), and short (S) TCF4 isoforms with minimal background. **B**: TCF4 immunostaining in 2-week-old macaque brain using ab217668. **B1**, shows that TCF4 staining concentrates in nuclei with minimal cytoplasmic or neuropil background. The arrow in **B1** indicates the nucleus shown in **B2–4**. **B2–4**, higher magnification reveals a punctate nuclear staining pattern, with little to no signal in condensed DNA regions (arrowheads) and the nucleolus (arrow). **C,D**: Validation via shRNA-mediated Tcf4 knockdown in cultured mouse cortical neurons. **C**: Control: robust nuclear TCF4 staining in untransfected and scrambled shRNA-transfected neurons (arrowhead). **D**: Tcf4-targeting shRNA: absence of TCF4 staining in transfected neurons (arrowhead). Scale bars: **B1**: 25 μm; **B2,3**: 5 μm; **C,D** upper panel: 50 μm; **C,D** lower panel: 10 μm.

We used short hairpin RNA (shRNA) knockdown to confirm the specificity of the rabbit monoclonal antibody (clone NCI-R159-6, Abcam Cat# ab217668, RRID:AB_2714172) used for this study (Figure 1). Briefly, cortical tissue from E15.5 wild-type C57BL/6J mice was dissected in Leibovitz’s L-15 medium, rinsed in Hanks’s balanced salt solution (HBSS), and dissociated with papain/DNase I in HBSS (30 min, 37°C). Papain was deactivated with Neurobasal Plus medium containing 5% FBS, and the tissue was gently triturated. Cells were washed in HBSS via centrifugation, re-suspended in a medium containing Neurobasal Plus medium with 5% fetal bovine serum (A525680; Gibco), GlutaMax (35050–061, Invitrogen), B27 Plus (A3582801, ThermoFisher), and Antibiotic-Antimycotic (15240–062, Invitrogen), and plated on poly-D-lysine coated coverslips (GG-12-PDL, Neuvitro) in a 24-well tissue culture plate at 250,000 cells per well. Half the medium was replaced every 3 days with Neurobasal Plus, GlutaMax, B27 Plus, and 2.46 μg/mL anti-mitotic agent 5-fluoro-2 ´-deoxyuridine (F0503, Millipore Sigma). On DIV3 (3 days *in vitro*), neurons were transfected with 0.33 μg CamKIIα-tdTomato plasmid (Mabb et al., 2016) together with 0.66 μg of vector expressing scramble shRNA (SHC002) or a mixture of five Tcf4-targeting shRNA vectors (TRCN0000012093 - TRCN0000012097) developed by the RNAi Consortium (TRC; Broad Institute) using Lipofectamine2000 reagent (11668019, Invitrogen) at 2:1 ratio per well. The cell plate was centrifuged in a 37°C preheated centrifuge at 1000 g for 3 min. One hour later, the media was removed and replaced with conditioned media. On DIV10, cells were briefly washed with PBS and then fixed with 4% paraformaldehyde (pH 7.4) in PBS at room temperature (RT) for 10 min, followed by PBS washes. Fixed cells were permeabilized and blocked simultaneously using PBS with 1% BSA and 0.1% Triton-X-100 (30 min, room temperature). Primary antibody incubation (TCF4, clone NCI-R159-6, Abcam Cat# ab217668, RRID:AB_2714172, at 1:250) was performed overnight in the same blocking solution at room temperature. Cells were washed 3 times in PBS (10 min each), followed by an overnight incubation with the secondary antibody. After three more washes in PBS (10 min each), coverslips were mounted using Vectashield.

### 2.5 Immunohistochemistry

Free-floating sections were treated with 3% H_2_O_2_ in PBS (0.1 M, pH 7.4) for 30 min to remove endogenous peroxidase. After blocking with 1% BSA in PBS, sections were incubated overnight at room temperature with primary antibody. The antibody used for staining was selected based on an initial trial, which is described at the beginning of the Results section. Specifically, we used a rabbit monoclonal antibody (clone NCI-R159-6, Abcam Cat# ab217668, RRID: AB_2714172) at a dilution of 1:1,000. Sections were then incubated with biotinylated secondary antibody (1:400) for 3 h, followed by ExtrAvidin-peroxidase complex (1:5,000) for 1 h. Peroxidase was visualized using a nickel-intensified diaminobenzidine (DAB) solution. This solution consisted of 0.05% DAB, 0.04% Nickel Ammonium Sulfate, 0.004% ammonium chloride, and 0.015% H_2_O_2_ in PBS, pH 7.2. Processed sections were mounted, air-dried, cleared with xylene, and coverslipped with D.P.X. mounting medium. Sections were analyzed using a Nikon Eclipse Ti2 microscope and scanned using a Slideview VS200 slide scanner (Olympus, Hamburg, Germany). Analysis was conducted using the QuPath software package (Bankhead et al., 2017).

## 3 Results

Previously, we used a green fluorescent protein reporter mouse to study TCF4 expression in the postnatal mouse brain (Kim et al., 2020), as we had yet to optimize our protocols using a TCF4 antibody. In this study, we complement that approach by employing classic DAB immunohistochemistry, and, importantly, we extend our investigation to the developing macaque brain. DAB immunohistochemistry allows direct visualization of the TCF4 protein, enhancing regional resolution and enabling a more accurate comparison between the mouse and macaque brain. However, the quality of immunohistochemistry data is only as good as the antibody used. To address this, we first evaluated seven TCF4 antibodies (Figure 1; Table 1; Supplementary Figures S1, S2). Santa Cruz Biotechnology antibody sc-393407 has previously been validated to detect specific TCF4 signals by Western blot analysis (Nurm et al., 2021) and to recognize three groups of TCF4 isoforms based on their molecular weight: long isoforms (TCF4-B^+/−^ and -C^+/−^), medium isoforms (TCF4-D^+/−^), and short isoforms (TCF4-A^+/−^ and -I^+/−^) (Supplementary Figure S1A) (Sirp et al., 2022). However, our experiments found it unsuitable for immunohistochemistry due to significant non-specific binding (Supplementary Figure S1B). We also do not recommend Sigma-Aldrich antibodies HPA025958 (Supplementary Figures S1C,D) and SAB1412620 (Supplementary Figures 1E,F) or LSBio antibodies LS-B8267 (Supplementary Figures 2C,D) and LS-C331289 (Supplementary Figures 2E,F) due to their non-specific labeling in both immunohistochemistry and Western blot applications (Supplementary Figures S1,S2). Abcam antibody ab130014 was suitable for mouse brain tissue analysis but ineffective for macaque brain tissue and Western blot analysis due to high background noise and overlapping non-specific bands (Supplementary Figures 2A,B). Ultimately, both Western blot and immunohistochemistry analyses identified the rabbit monoclonal antibody clone NCI-R159-6 (Abcam Cat# ab217668, RRID: AB_2714172) as the most effective for this study. It showed strong nuclear staining in both mouse and macaque brains and provided a clear, strong specific signal for long, medium, and short TCF4 isoforms with minimal background noise in Western blot analysis (Figure 1A). In agreement with its role as a transcription factor, TCF4 immunostaining was detected exclusively in cell nuclei (Figure 1B) with minimal to no background staining present in the cytoplasm and neuropil. TCF4 staining appeared as discrete puncta (Figure 1B). These puncta were distributed among DAPI “hotspots” (arrowheads in Figure 1B4) but did not coincide with them. Similarly, TCF4 staining appeared absent from nucleoli (arrow in Figure 1B4). To confirm its specificity, we employed shRNA knockdown. As shown in Figure 1C, TCF4 staining was robust in the nuclei of untransfected and scrambled shRNA-transfected cells but absent in TCF4-shRNA-transfected cells (Figure 1D), confirming the antibody’s specificity. Consequently, this antibody was used for all subsequent experiments.

**Table 1:** Details of commercial TCF4 antibodies evaluated in this study.

Company	Catalogue	Epitopes	Species	IHC	WB
Abcam	ab217668	Central region^1^	Rabbit monoclonal	Strong nuclear staining in both mouse and macaque brains.	Clear, strong specific signal of long, medium, and short TCF4 isoforms with minimal background noise.
Abcam	ab130014	Mouse aa 50–150	Rabbit polyclonal	Weak nuclear staining in mouse brains. Strong cytoplasmic staining in macaque brains.	High background noise and overlapping unspecific band, making specific signal detection difficult.
LSBio	LS-B8267	Human N-terminus	Rabbit polyclonal	Faint neuropil staining in both mouse and macaque.	Multiple unspecific bands, obscuring the specific signal.
LSBio	LS-C331289	Human aa 400–500	Rabbit polyclonal	Cytoplasmic and dendritic staining in both species	Multiple unspecific bands, obscuring the specific signal.
Santa Cruz Biotechnology	sc-393407	Human aa 251–278	Mouse monoclonal	Cytoplasmic and dendritic staining in both mouse and macaque brains.	Clear, strong specific signal of long, medium, and short TCF4 isoforms with minimal background noise.
Sigma Aldrich	HPA025958	Human recombinant fragment	Rabbit polyclonal	Cytoplasmic and dendritic staining in both mouse and macaque brains.	Strong unspecific signal and weak isoform distinction.
Sigma Aldrich	SAB1412620	Human full-length recombinant	Mouse monoclonal	No detectable signal in P1 mice; nuclear and cytoplasmic staining in P90 mice and macaques.	Multiple unspecific bands, obscuring the specific signal.

^1^The exact immunogen sequence is proprietary. Abcam has confirmed that it targets a “central” region of the protein (i.e., not at the N or C terminal part), enabling the antibody to react with all TCF4 isoforms in mice, macaques, and humans (personal communication from Abcam to ACB). IHC, immunohistochemistry; WB, western blot.

### 3.1 Overall TCF4 expression in the developing mouse brain

**Figure 2:**
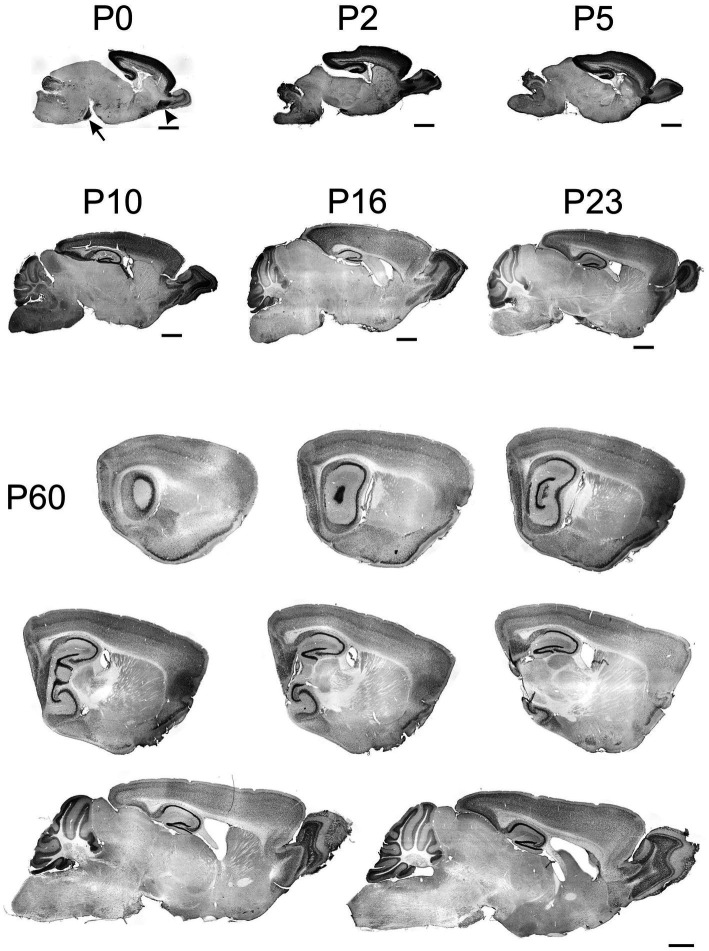
Immunoreactivity to TCF4 in sagittal mouse brain sections at P0 through P60. From P0 to P60, TCF4 expression is primarily seen in the cortical plate and cerebellum. Initially concentrating in the hippocampal formation and isocortex, with a notable presence in the anterior olfactory nucleus (arrowhead) and pontine grey (arrow). TCF4 expression peaks around P2–P5. After that, it gradually decreases across all regions, stabilizing by P60 with the highest levels remaining in the cerebellum, hippocampal formation, cerebral cortex, and olfactory bulb. Scale bars = 1 mm.

TCF4 expression in the developing mouse brain was examined from birth (P0) to adulthood (P60). TCF4 was predominantly expressed throughout this period in the cortical plate (hippocampal formation, cerebral cortex, olfactory bulb) and the cerebellum (Figure 2). At P0, TCF4 was concentrated in the hippocampal formation and isocortex, with notable staining in the anterior olfactory nucleus (arrowhead in Figure 2) and pontine grey (arrow in Figure 2). Lower levels were seen in the cerebellum, with sparse staining in other brain regions. By P2, the overall distribution remained similar, but staining intensified in the cerebellum, olfactory bulb, and pons. At P5, TCF4 staining began to decline in the cerebellum and olfactory bulb. From P10 onward, TCF4 expression gradually decreased in all brain regions, reaching stable adult levels by P60. Staining remained most prominent at this stage in the cerebellum, hippocampal formation, cerebral cortex, and olfactory bulb.

### 3.2 Regional TCF4 expression in the developing mouse brain

**Figure 3:**
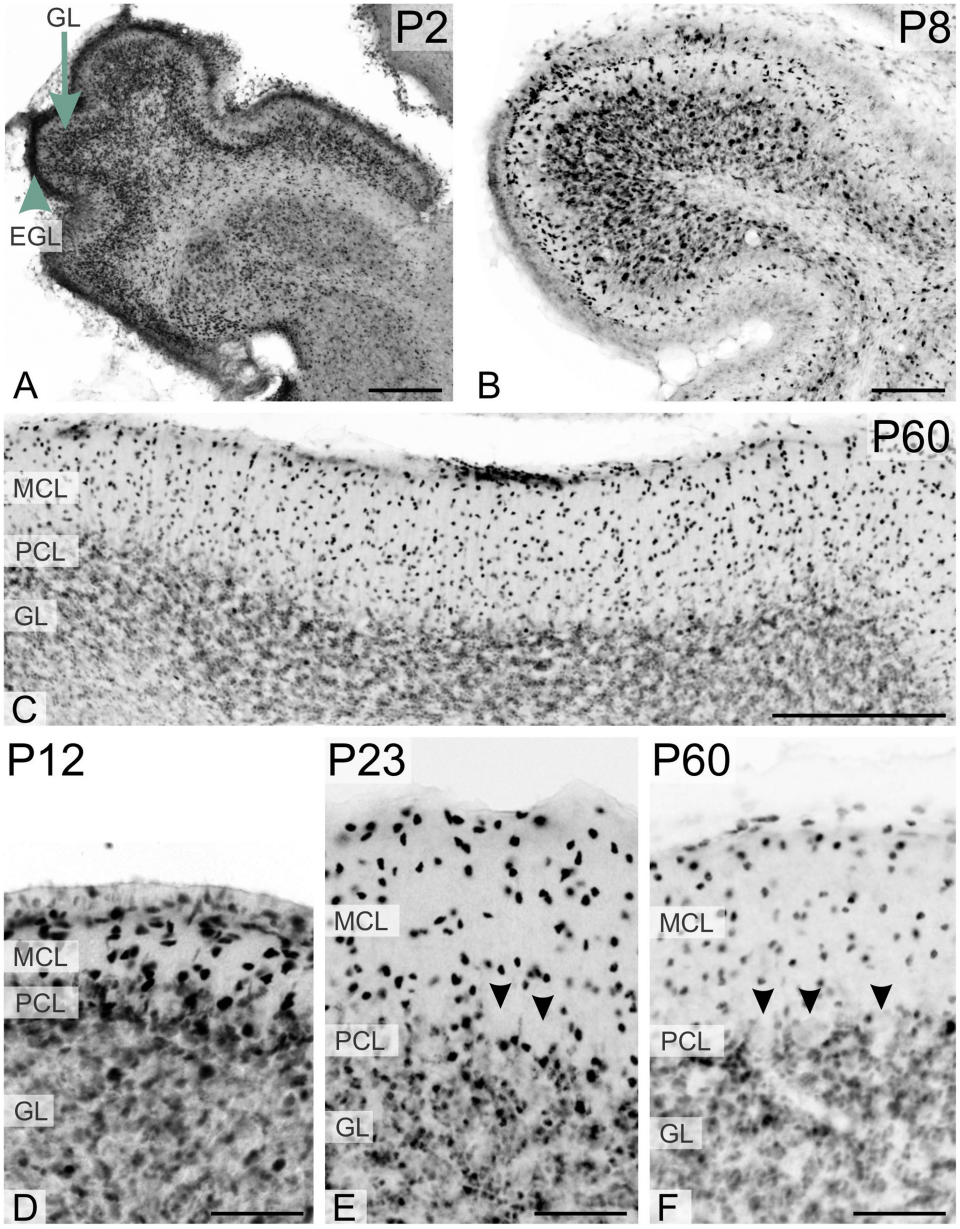
Immunoreactivity to TCF4 in the developing mouse cerebellum. At P2, TCF4 is highly expressed in the external (arrowhead in **A**) and internal granular layers (arrow in **A**). Over time, expression decreases in the granule cell layer but increases in the molecular layer. Small cells in the Purkinje cell layer remain TCF4-positive throughout, while Purkinje cells are consistently TCF4-negative (arrowheads in **E,F**). GL, granule cell layer; EGL, external granular layer; MCL, molecular cell layer; PCL, Purkinje cell layer. Scale bars: **A–C**: 200 μm; **D–F**: 50 μm.

**Figure 4:**
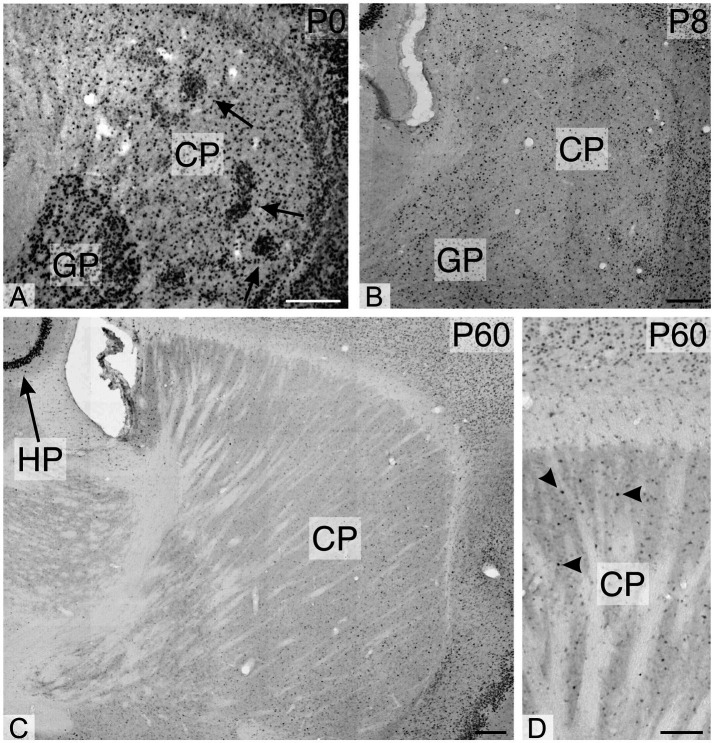
Immunoreactivity to TCF4 in the developing mouse striatum and pallidum. TCF4 immunoreactivity is prominent in dense cell clusters (arrows in **A**) and dispersed cells within the pallidum and caudoputamen. TCF4 expression diminishes rapidly during postnatal development. By P60, most cells in these regions exhibit weak TCF4 immunoreactivity, with only a few cells showing strong positivity (arrowheads in **D**), albeit less intense than in the hippocampus. The arrow in C highlights the marked difference in staining intensity between the pyramidal cell layer of the hippocampal region and the adjacent caudoputamen. CP, caudoputamen; GP, globus pallidus; HP, hippocampus. Scale bars: **A–C**: 200 μm; D: 100 μm.

During early postnatal development of the cerebellum, TCF4 was strongly expressed in the external granular layer (arrowhead in Figure 3A) and the granule cell layer (arrow in Figure 3A). As postnatal development progressed, staining in the granule cell layer diminished, while strongly positive cells appeared in the molecular layer (Figures 3E,F). At all stages, small cells in the Purkinje cell layer were immunopositive, but Purkinje cells themselves were never TCF4-positive (arrowheads pointing to ghosts of Purkinje cells in Figures 3E,F). At birth, many strongly TCF4-positive cells were seen in the striatum and pallidum (Figure 4A). In the caudate and putamen, clusters of tightly packed cells were immunopositive (arrows in Figure 4A), along with scattered cells. The density of TCF4-positive cells quickly decreased as development progressed, and by adulthood, most cells in the striatum and pallidum were only weakly positive (arrowheads in Figure 4D). A small number of scattered cells was still clearly immunopositive, although not as strongly as cells in the hippocampus (arrow in Figure 4C).

**Figure 5:**
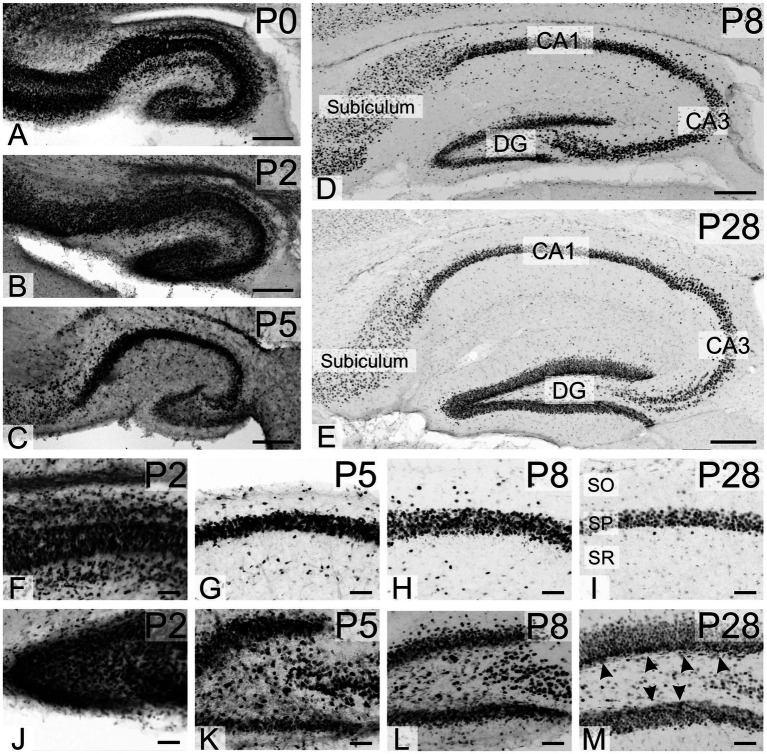
Immunoreactivity to TCF4 in the developing mouse hippocampal formation. **A–E**: overview of TCF4 expression in the developing hippocampus. **F–I**: closeup of the CA1 region. **J–M**: closeup of the dentate gyrus. P0–P2 hippocampal formation shows strong TCF4 expression throughout, especially in immature pyramidal cell and dentate gyrus granule cell layers. Expression peaks around P8, resembling mature patterns. At P28, TCF4 levels decrease but are still notable. Interestingly, small cells in the dentate gyrus sub-granular zone maintain high expression (arrowheads in **M**), possibly representing precursor cells or immature neurons. CA1, CA3, fields of the hippocampus, DG, dentate gyrus; SO, stratum oriens; SP, stratum pyramidale; SR, stratum radiatum. Scale bars: **A**–**E**: 200 μm; **F**–**M**: 50 μm.

At P0 and P2, strong TCF4 staining was seen in all hippocampal regions, particularly the still immature pyramidal cell layer and the granule cell layer of the dentate gyrus (Figures 5A,B). By P8, TCF4 expression in the hippocampal formation decreased to the levels seen in the mature brain, with strong staining in the pyramidal cell layer and dentate gyrus granule cell layer (Figure 5D). By P20-P23, TCF4 expression levels had decreased, although were still strong, in all regions, stabilizing at adult levels. Notably, a narrow layer of small cells in the sub-granular zone of the dentate gyrus maintained early postnatal expression levels (arrowheads in Figure 5M). Based on their size, shape, and location, we speculate that they represent precursor cells and immature granule cells.

**Figure 6:**
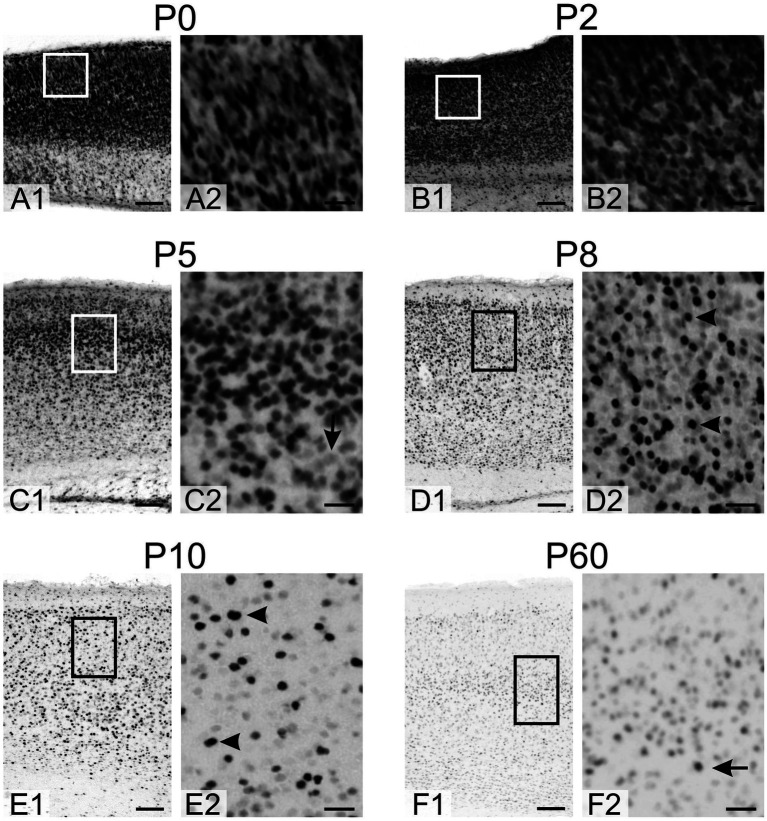
Immunoreactivity to TCF4 in the developing mouse neocortex. **A1**–**F1**: Overview of TCF4 expression across the neocortex layers. **A2**–**F2**: Magnified view of the boxed region in **A1**–**F1**. At P0 and P2, all neocortical layers exhibit densely packed, strongly TCF4-positive cells. By P5, TCF4-positive cell density decreases, particularly in the more mature deep layers, with the emergence of cells showing lower TCF4 immunoreactivity (arrow in **C2**). At P8 and P10, strongly positive cells (arrowheads in **D2**, **E2**) continue to decline in number, while lightly stained cells become more prevalent as the neocortex matures. By P60, TCF4 expression is markedly reduced overall, with only a small subset of cells remaining strongly positive (arrow in **F2**) amidst a larger population of weakly positive cells. Scale bars: **A1–F1**: 100 μm; **A2**–**F2**: 25 μm.

At P0 and P2, densely packed, strongly positive cells were seen in all neocortical layers (Figure 6). By P5, the density of TCF4-positive cells decreased, particularly in the more mature deeper layers, and cells with lower TCF4 staining began to appear. At P8 and P10, the density of strongly positive cells (arrowheads in Figures 6D2,E2) continued to decrease, while the number of lightly stained cells increased as the neocortex matured. By P60, overall TCF4 expression was significantly reduced, with only a small population of positive cells among a larger population of weakly positive cells (arrow in Figures 6F2).

**Figure 7:**
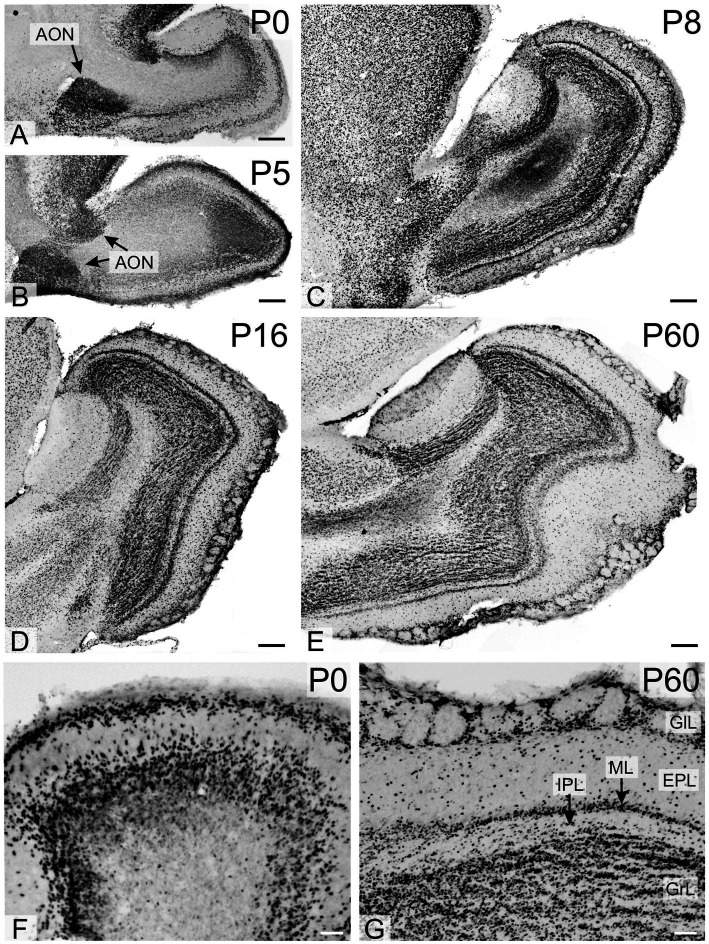
Immunoreactivity to TCF4 in the developing mouse olfactory bulb. High TCF4 expression in the anterior olfactory nucleus, olfactory bulb glomerular layer, and developing internal plexus layer are seen at P0-P5. By P8, TCF4 staining in the olfactory bulb reached adult-like patterns, with strongly positive cells across all layers. AON, anterior olfactory nucleus; EPL, external plexiform layer; GlL, glomerular layer; GrL, Granular layer; IPL, internal plexiform layer; ML, mitral cell layer. Scale bars: **A–E**: 200 μm; **F,G**: 50 μm.

At P0 and P2, a dense population of strongly positive cells was present in the anterior olfactory nucleus (arrows in Figures 7A,B), with strongly positive cells also seen in the olfactory bulb, particularly in the glomerular layer. By P16, TCF4 staining in the olfactory bulb resembled that of the adult brain, with strongly positive cells in all layers (Figure 7).

### 3.3 TCF4 expression in the developing macaque brain

**Figure 8:**
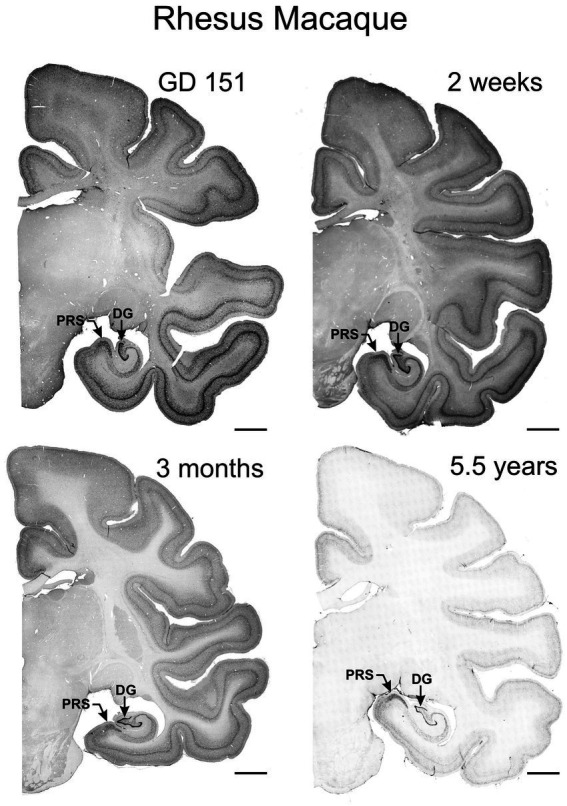
Immunoreactivity to TCF4 in the prenatal, infant, and young adult rhesus macaque brain. TCF4 staining in coronal sections from gestation day 151, 2-week-old, 3-month-old, and 5.5-year-old rhesus macaque brain. TCF4 expression is widespread throughout the brain, peaking prenatally and decreasing with age. It concentrates in the grey matter, with the strongest expression in the presubiculum and dentate gyrus of the hippocampal formation and neocortical layers II and IV. In contrast, the thalamus shows relatively lower levels of TCF4. DG, dentate gyrus; PRS, presubiculum; Scale bar: 0.5 cm.

**Figure 9:**
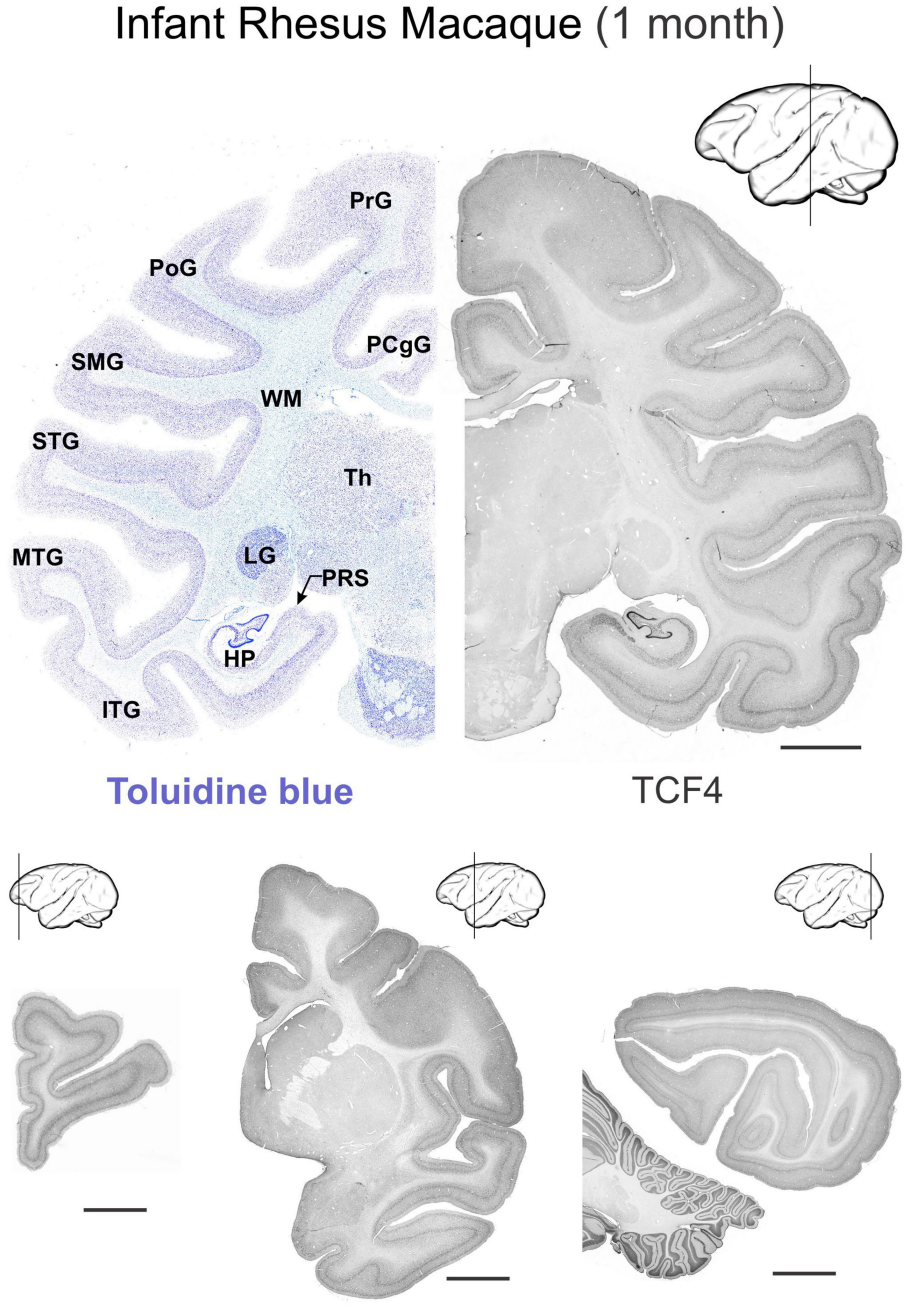
Immunoreactivity to TCF4 in the infant (1 month) rhesus macaque brain. TCF4 expression in 4 coronal sections across the 1-month-old rhesus macaque brain. Staining is stronger in grey matter than white matter, with the cerebellum, hippocampal formation, and neocortex showing the highest expression throughout development. Subcortical regions show lower expression levels. Scale bars: 0.5 cm. HP, hippocampal formation (dentate gyrus, hippocampus, subiculum, presubiculum, parasubiculum); ITG, inferior temporal gyrus; LG, lateral geniculate nucleus; MTG, middle temporal gyrus; PCgG, posterior cingulate gyrus; PoG, postcentral gyrus; PrG, precentral gyrus; PRS, presubiculum; SMG, supramarginal gyrus; STG, superior temporal gyrus; Th, thalamus; WM, white matter. Scale bar: 0.5 cm.

We studied TCF4 regional distribution in normal prenatal (GD 151), neonatal (2 and 4 weeks old), infant (3 months), and young adult (5.5 years) rhesus macaque brains. Mirroring the pattern seen in mice, TCF4 expression was widespread throughout the brain at all developmental stages but strongest prenatally, gradually decreasing with age (Figures 8, 9). Immunostaining was concentrated in the grey matter, with weaker staining in the white matter. Consistent with the mouse brain, TCF4 staining was most prominent in the cerebellum, hippocampal formation, and neocortex during prenatal development and into adulthood. Conversely, subcortical structures showed lower expression levels.

**Figure 10:**
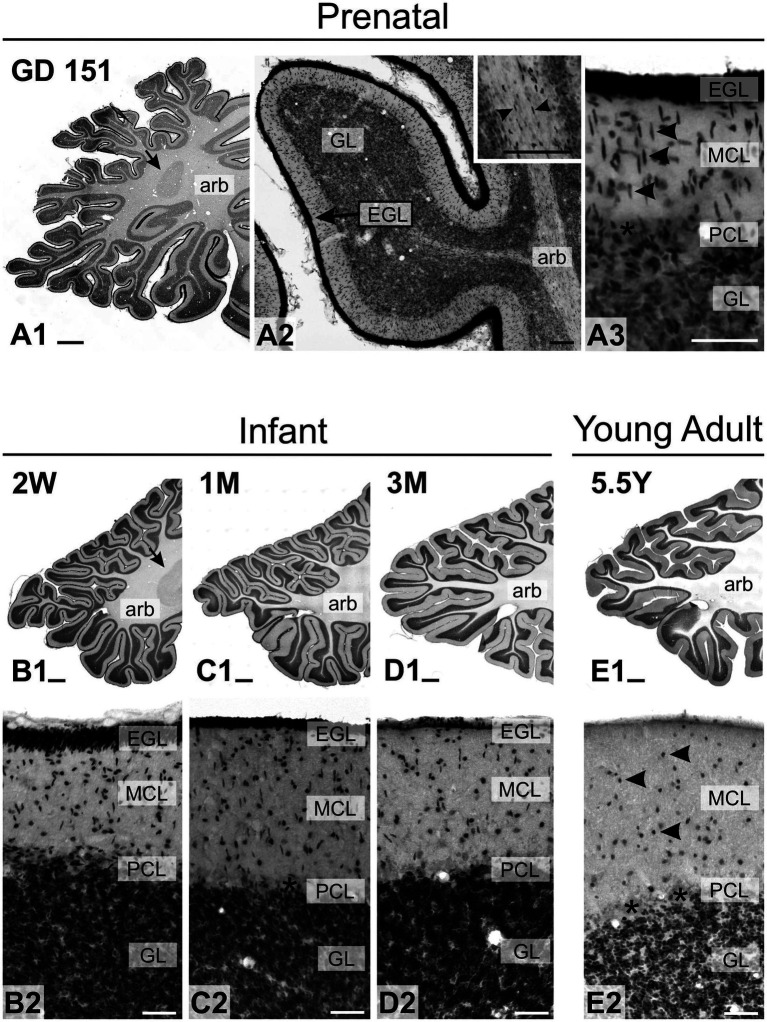
Immunoreactivity to TCF4 in the developing macaque cerebellum. TCF4 immunoreactivity in the developing macaque cerebellum is consistently high, peaking prenatally. Intense staining is observed in granule cell precursors within the external granule cell layer (GD 151 to 3 months), migrating postmitotic granule cells with distinctive oblong shapes (arrowheads in **A3**), and mature granule cells in the granule cell layer. Deep cerebellar nuclei exhibit only weak staining (arrows in **A1,B1**). Small oblong immunopositive cells are visible in the arbor vitae from prenatal stages to 4 weeks of age (arrowheads in inset **A2**). During adolescence, granule cells maintain TCF4 immunoreactivity at reduced intensity, while scattered small immunopositive cells appear in the molecular layer (arrowheads in **E2**). Purkinje cells consistently lack TCF4 immunoreactivity across all ages studied (asterisks). abr: arbor vitae; EGL: External granule layer, GL: Granule cell layer, MCL: Molecular cell layer, PCL: Purkinje cell layer. Scale bars: **A1**–**E1**: 1 mm; **A3**: 100 μm; **B3**, **B2**–**E2**: 100 μm.

**Figure 11:**
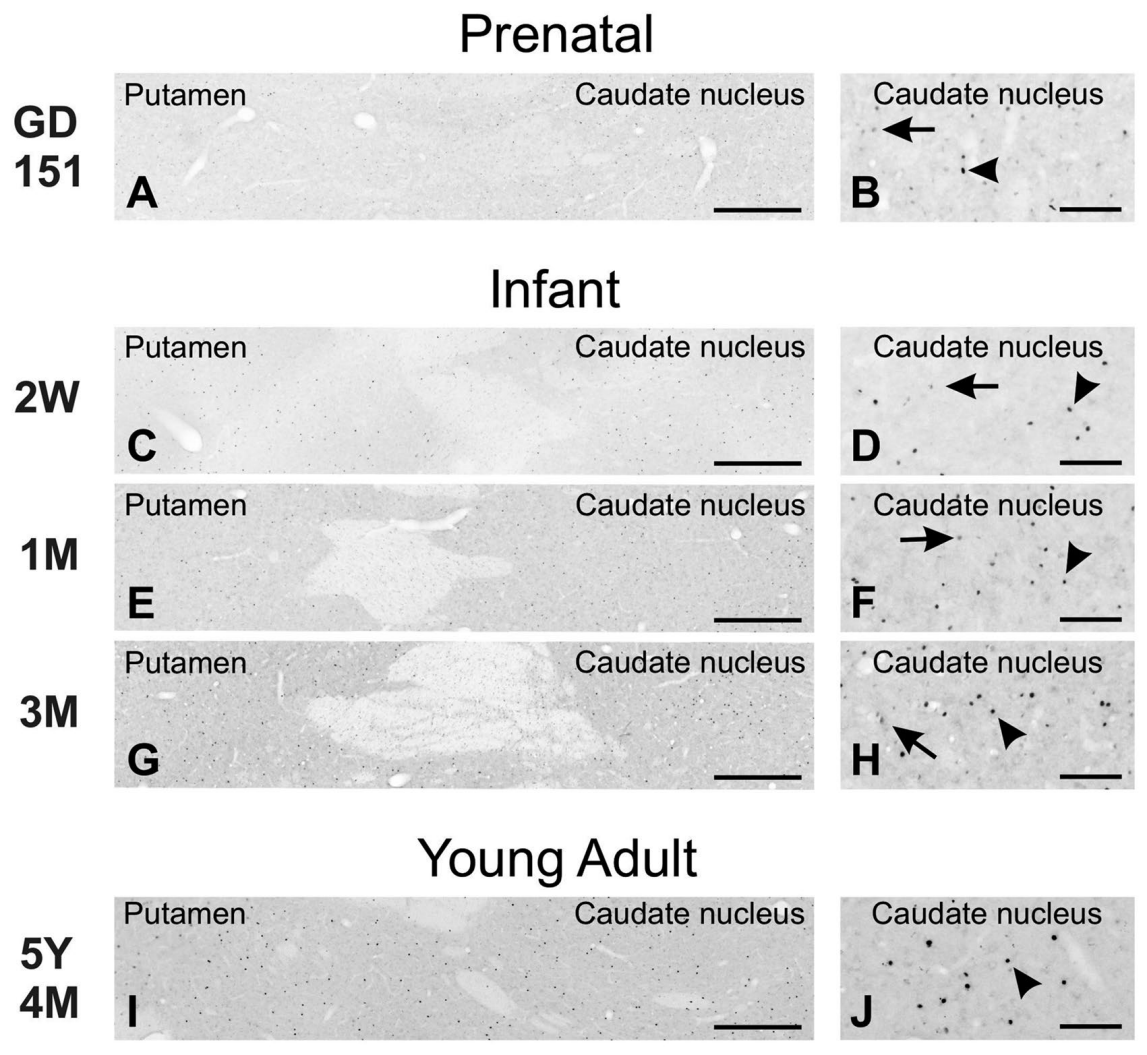
Immunoreactivity to TCF4 in the developing macaque striatum. Prenatally, rare, strongly TCF4-positive cells (arrowheads) are scattered among many weakly stained cells (arrows). The number of strongly positive cells increases during development, forming a distinct, strongly TCF4-positive population (H and J). Scale bars: **A,C,E,G,I**: 500 μm; **B,D,F,H,J**: 100 μm.

As in the mouse brain, TCF4 expression was consistently high in the cerebellum across all ages examined. Still, it was strongest prenatally (Figure 10). This strong expression was attributed to intense staining in granule cell precursors located in the external granule cell layer (GD 151 to 3-month-old) and mature granule cells located in the granule cell layer (2 weeks old onward). In prenatal and early postnatal (up to 2 months old) brains, proliferating granule cell progenitors in the external granular layer showed strong immunopositivity. Migrating postmitotic granule cells, characterized by their distinctive oblong shapes (arrowheads in Figure 10A3), were also strongly labeled as they moved from the external granular cell layer toward the Purkinje cell layer. Mature granule cells within the granule cell layer were similarly positive for TCF4 (Figures 10B–E). While this staining persisted in granule cells throughout adolescence, it was less intense. Scattered small round cells were stained in the molecular layer (arrowheads in Figure 10E2). Purkinje cells consistently lacked TCF4 immunoreactivity across all ages studied (asterisk on Purkinje cell ghosts in Figures 10A3,C,E). Within the arbor vitae, small oblong cells, possibly postmitotic Golgi cells migrating towards the developing granular layer (Komuro and Rakic, 1998), were immunopositive from prenatal stages to 4 weeks of age (arrowheads in inset in Figure 10A2). Tiny cells were scattered throughout the white matter at all ages. In contrast, TCF4 staining in the cerebellar nuclei was low (arrows in Figure 10), limited to a few scattered small, immunopositive cells. Projection neurons displayed weak staining, barely distinguishable from background levels (data not shown). After the cerebellum, the strongest concentration of TCF4 immunopositive cells was seen in the cerebrum in the isocortex and hippocampal formation. In the striatum and pallidum, TCF4 was seen in a small set of medium-sized cells (Figure 11). Prenatally, very few strongly immunopositive cells (arrowheads in Figure 11) were scattered among a larger population of barely stained cells (arrows in Figure 11). As brain development progressed, the number of strongly positive cells increased, forming a well-defined population of TCF4-expressing cells (Figure 11J). This pattern differs from that seen in the mouse brain, where TCF4 expression in the striatum was initially high in early postnatal stages but quickly declined, resulting in a small group of weakly stained cells.

**Figure 12:**
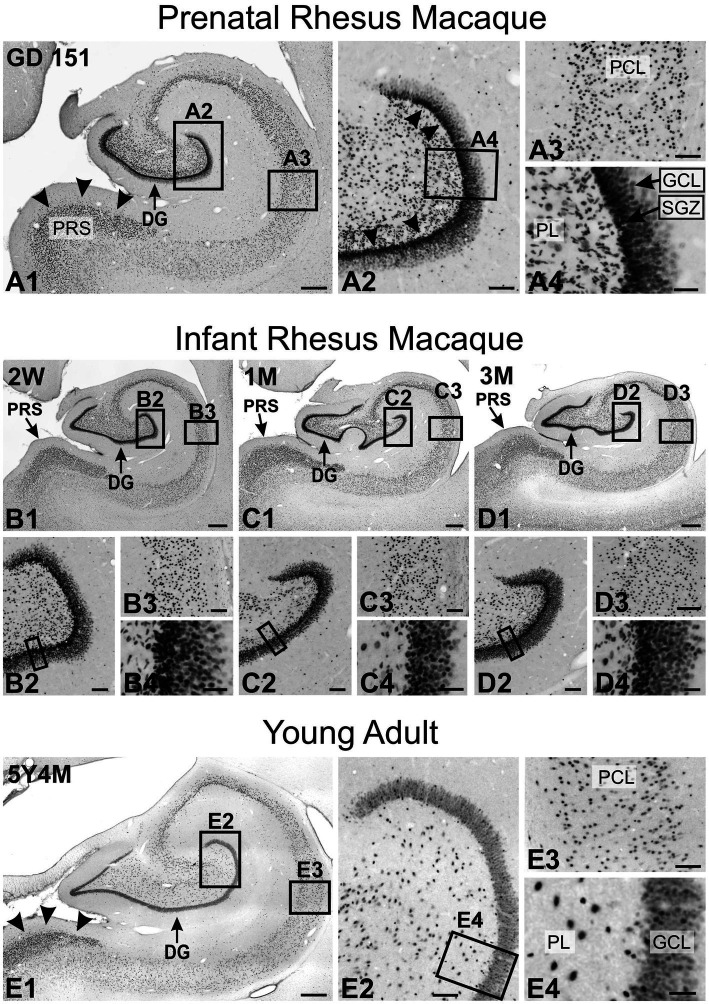
Immunoreactivity to TCF4 in the developing macaque hippocampal formation. **A1**: Prenatally, the most prominent staining is observed in the dentate gyrus, followed by layer II of the presubiculum (arrowheads). **A2** is a magnified view of the dentate gyrus region. The most intense staining is seen in the neuronal progenitor cells of the subgranular zone (arrowheads), followed by cells in the polymorphic layer and granule cells in the granule cell layer. **A3** is a magnified view of the pyramidal cell layer. Most pyramidal neurons show strong TCF4 staining. **A4** is a magnified view of the dentate gyrus polymorphic and granule cell layers. Intense TCF4 expression is seen in the cells within the subgranular zone and the elongated migrating cells in the polymorphic layer. **B1–D1** are overviews of TCF4 expression in the still-developing infant hippocampus. **B2–D2** are magnified views of the dentate gyrus. **B3–D3** are magnified views of the pyramidal cell layer. **B4–D4** are magnifications of the dentate gyrus polymorphic and granule cell layers. TCF4 staining in the infant hippocampus is similar to that seen prenatally. **E1** is an overview of TCF4 expression in the young adult hippocampus. TCF4 staining is less than in the infant hippocampus but is still robust, especially in the presubiculum (arrowheads) and the dentate gyrus. **E2** is a magnified view of the dentate gyrus. A small population of cells maintained high TCF4 levels, with more such cells in the polymorphic layer than in the granule cell layer. Other cells have a much lower but still robust TCF4 expression. **E3** is a magnified view of the pyramidal cell layer. Most pyramidal neurons show robust TCF4 staining, although less intense than in the infant brain. **E4** is a magnified view of the dentate gyrus polymorphic and granule cell layers. Most granule cells are robustly stained for TCF4, and a small population of granule cell are more strongly stained, as were cells in the polymorphic layer. DG, dentate gyrus; GCL, granule cell layer; PCL, pyramidal cell layer; PL, polymorphic layer; PRS, presubiculum; SGZ, subgranular zone. Scale bars: **A1–E1**: 500 μm; **A2–E2**: 100 μm; **A3–E3**: 100 μm; **A4–E4**: 40 μm.

TCF4 exhibited strong expression in the hippocampal formation (Figure 12). Prenatally, the most prominent staining was observed in the dentate gyrus, mirroring patterns in the mouse brain. Strong staining was evident in the neuronal progenitor cells of the subgranular zone (arrowheads in Figure 12A2), in small, oblong, migrating cells within the polymorphic cell layer (Figure 12A4), and in dentate granule cells (Figure 12A4). The pyramidal cell layer of the hippocampus also showed robust TCF4 staining (Figure 12A3). Notably, staining was particularly intense in the small pyramidal cells constituting layer II of the presubiculum (arrowheads in Figure 12A1). This prominent staining persisted in the young adult animal (Figures 9, 12B–D). As the hippocampus matured, TCF4 staining generally decreased, showing more moderate nuclear staining across all hippocampal regions in the young adult brain. However, staining was still robust, particularly in the presubiculum (arrowheads in Figure 12E1). In the mature dentate gyrus, only a small, seemingly random population of cells maintained high TCF4 levels, with more such cells in the polymorphic layer than in the granule cell layer (Figure 12E4). This pattern differs from adult mice, where the dentate gyrus subgranular zone maintained high TCF4 expression (compare Figure 12E4 with Figure 5M). Similarly, in the pyramidal cell layer, only a small subpopulation of cells remained strongly labeled for TCF4 (Figure 12E3).

**Figure 13:**
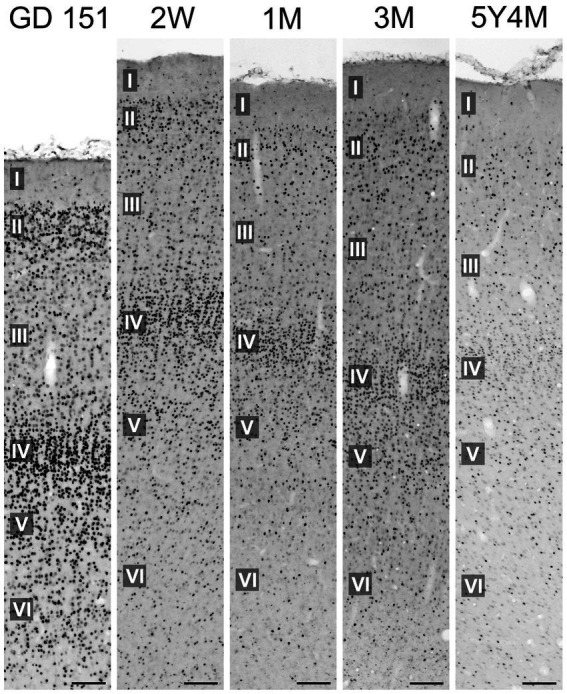
Immunoreactivity to TCF4 in the developing macaque inferior temporal gyrus. At GD 151, neocortical TCF4 expression concentrates in layers II and IV. As the brain matures, the overall number of TCF4-positive cells decreases. However, in the young adult neocortex, a subset of cells continues to exhibit strong TCF4 staining. Scale bars: 50 μm.

At GD 151, the neocortex showed a distinct pattern of TCF4 expression, with staining concentrated in layers II and IV (Figure 13). This two-layer pattern was notably prominent in the temporal lobe cortex but absent in the motor cortex. This was in striking contrast to the mouse neocortex which displayed a more uniform TCF4 distribution, mostly devoid of this distinct layered pattern. As the neocortex matures, TCF4 staining gradually decreased. In the young adult brain, a smaller population of cells maintained strong TCF4 expression similar to the situation in the hippocampal formation.

## 4 Discussion

TCF4 is implicated in a broad spectrum of cognitive disorders (Del-Favero et al., 2002; Amiel et al., 2007; Brockschmidt et al., 2007; Zweier et al., 2007; Stefansson et al., 2009; Schizophrenia Psychiatric Genome-Wide Association Study Consortium, 2011; Cross-Disorder Group of the Psychiatric Genomics Consortium, 2013; Wray et al., 2018; Gelernter et al., 2019; Teixeira et al., 2021). Common genetic variants are associated with conditions such as schizophrenia, while rare variants have been found in individuals with intellectual disability. TCF4 haploinsufficiency causes Pitt-Hopkins syndrome (Chen et al., 2021). Mouse models have been instrumental in our understanding of TCF4 biology and its role in disorders (Chen et al., 2023b; Espinoza et al., 2024). These models have proven essential for characterizing disease pathology and evaluating potential therapeutic interventions (Kim et al., 2022; Bohlen et al., 2023; Martinowich et al., 2023;Dennys et al., 2024). Mouse models, however, cannot fully recapitulate the complex neuropsychiatric phenotypes that affect core human traits like intelligence, language, and social behavior. This limitation stems from ~90 million years of evolutionary divergence between mice and humans (Kumar et al., 2017), which has led to significant differences in gene expression, neuroanatomy, and complex behaviors between the two species (Belmonte et al., 2015; Kaiser and Feng, 2015; Jennings et al., 2016). Transcriptomic analyses further underscore these disparities, revealing substantial differences in molecular signatures, particularly in genes associated with neurological disorders (Parikshak et al., 2015; Pembroke et al., 2021). Studying TCF4 in the macaque brain, a closer relative to humans, helps bridge the gap between rodent and human research. In this study, we provide the first detailed characterization of TCF4 expression in developing primate brains and compare it to its expression in the developing mouse brain. A limitation of this study is the need for more precise knowledge of the specific TCF4 isoforms recognized by the antibody used. While the antibody’s antigen target suggests it may recognize all isoforms, it is possible that it does not bind them with equal affinity, potentially introducing bias in the observed staining patterns. However, the TCF4 distribution observed in the mouse brain in this study is consistent with previous findings obtained through other methods, including northern blot analysis, *in situ* hybridization, reverse-transcription quantitative PCR, and a green fluorescent protein reporter of TCF4 expression (Soosaar et al., 1994; Ravanpay and Olson, 2008; Li et al., 2019; Kim et al., 2020; Phan et al., 2020). This provides confidence in the validity of our results.

The cellular expression of TCF4 in the macaque brain aligns well with the understanding of its function gained from mouse studies. For instance, the most robust expression is seen in neurogenic zones, such as the cerebellar cortex’s external granular layer and the dentate gyrus’s subgranular zone, supporting a potential role for TCF4 in neurogenesis. Moreover, strong TCF4 expression in migrating granule cells is consistent with its involvement in cell migration. Importantly, significant TCF4 expression persists in the mature macaque brain, suggesting, as in mice, a role for TCF4 in the adult brain.

While the overall developmental trajectory of TCF4 expression is largely conserved between macaques and mice, accounting for species-specific differences in developmental timing and brain anatomy, close examination reveals several distinct differences. Key similarities between mice and macaques include the overall developmental regional distribution of TCF4. In both species, TCF4 is broadly expressed across the brain throughout development, with consistently higher levels seen in the cerebellum, hippocampal formation, and neocortex. Additionally, TCF4 expression levels decrease as the brain matures in both species but remain expressed significantly in the adult brain. As expected, yet still notable, TCF4 expression was highly concentrated in the nuclei of both species, consistent with its role as a transcription factor.

Close examination of several brain regions reveals interspecies differences in TCF4 expression patterns (Table 2). Whether these differences are idiosyncratic variations or reflect genuine evolutionary changes in TCF4 expression or function between mice and macaques is unclear. In both species, we saw high TCF4 expression in the subgranular zone of the dentate gyrus, where neurogenesis occurs (Kempermann et al., 2015). However, while this high expression persists into adulthood in mice, it is notably absent in the young adult macaque brain. Adult neurogenesis is well-established in rodents, but it remains controversial in primates (Jabes et al., 2010). Despite multiple studies reporting adult neurogenesis in the dentate gyrus of marmosets, macaques, and humans, a recent study suggests it may be limited to children and undetectable in adults (Gould et al., 1998; Gould et al., 1999; Gould, 2007; Spalding et al., 2013; Boldrini et al., 2018; Sorrells et al., 2018). Sorrells et al. (2018) also found that in the subgranular zone of macaque hippocampal formation proliferation is seen in the early postnatal period and diminishes during juvenile development, in line with earlier 3H-TdR autoradiographic method studies (Eckenhoff and Rakic, 1988). Therefore, the absence of high TCF4 expression in the adult macaque dentate gyrus likely reflects an idiosyncratic difference rather than a fundamental species-specific change in TCF4 expression patterns.

**Table 2:** Comparison of TCF4 expression patterns in developing mouse and macaque brains.

Brain Region	Mouse TCF4 Expression	Macaque TCF4 Expression
Subgranular zone (Dentate gyrus)	High expression in early development High expression persists into adulthood	High expression in early development Notably absent in young adult brain
Striatum	Robust expression at P0 Decreases but remains significant until ~P10 Rapid decrease after P10 Small population of weakly stained cells in adults	Few strongly TCF4-positive cells at GD 151 Population of immunoreactive cells increases as brain matures Staining remains in young adults
Neocortex	Largely absent layered pattern	Distinct layered pattern Notable concentration of TCF4-positive cells in layers 2 and 4

Interspecies differences in TCF4 expression were also seen in the striatum, a brain region crucial for goal-directed behavior, habit formation, learning, and value processing (Graybiel and Grafton, 2015; Cox and Witten, 2019). The striatum has also been implicated in various brain disorders, including schizophrenia (McCutcheon et al., 2019). TCF4 expression in the striatum was robust in the mouse brain at P0. It decreased but remained significant until approximately P10, followed by a rapid decrease to a small population of weakly stained cells. Conversely, in the macaque striatum, only a few cells were strongly TCF4 positive at GD 151. However, this population increased as the brain matured and remained present even in young adults. There are notable differences between the rodent and primate striatum (Balleine and O'Doherty, 2010; Woolley et al., 2013; Heilbronner et al., 2016). The primate striatum is divided into three distinct regions: the caudate nucleus, putamen, and ventral striatum. In contrast, the rodent striatum called the caudoputamen, is a single, unified structure. Notably, the anatomical connections of the rodent and primate striatum differ significantly. In rodents, five sensory inputs from the cortex and thalamus converge onto the striatum; in primates, this convergence is not seen. Instead, the primate striatum primarily receives inputs from visual areas. These differences and the likely associated circuitry variations (Lee et al., 2023) may underlie the observed differences in TCF4 expression between mice and macaques.

The most visually striking difference in TCF4 expression between species is the distinct layered pattern in the macaque neocortex, with a notable concentration of TCF4-positive cells in layers 2 and 4. This layered pattern is largely absent in the mouse brain. Neocortical expansion is a key feature of primate evolution, likely driven by primate-specific gene expression patterns that underlie more complex cognitive functions. Relevant to our results, primates have a larger and more complex cortical layer 4 which receives inputs from the thalamus compared to rodents (Cadwell et al., 2019). Recent cross-species transcriptomic analyses have also found primate-specific cell types enriched in layer 4 (Chen et al., 2023a). Given the importance of layer 4 in primate cortical organization and function, the observed differences in TCF4 expression in this layer are particularly intriguing and call for further investigation.

A precise understanding of TCF4’s cellular expression in the developing brain is crucial for developing effective and safe therapies for PTHS and other TCF4-related disorders. Our findings underscore the importance of combining rodent and primate studies to understand TCF4 function comprehensively. While the observed interspecies differences in TCF4 expression may appear subtle, they are nonetheless significant, especially considering how this might impact the design of future genetic interventions. These species differences may reflect evolutionary adaptations or genetic drift that have shaped the unique features of the primate brain, potentially contributing to its distinct cognitive abilities and susceptibility to human-specific cognitive disorders.

## Data Availability

The original contributions presented in the study are included in the article/Supplementary material, further inquiries can be directed to the corresponding authors.
