# Supplementary figures and images for: Transcription factor 4 expression in the developing non-human primate brain: a comparative analysis with the mouse brain
Source: Front Neuroanat. 2024 Oct 22;18:1478689. doi: 10.3389/fnana.2024.1478689 (PMC11534587; doi:10.3389/fnana.2024.1478689)

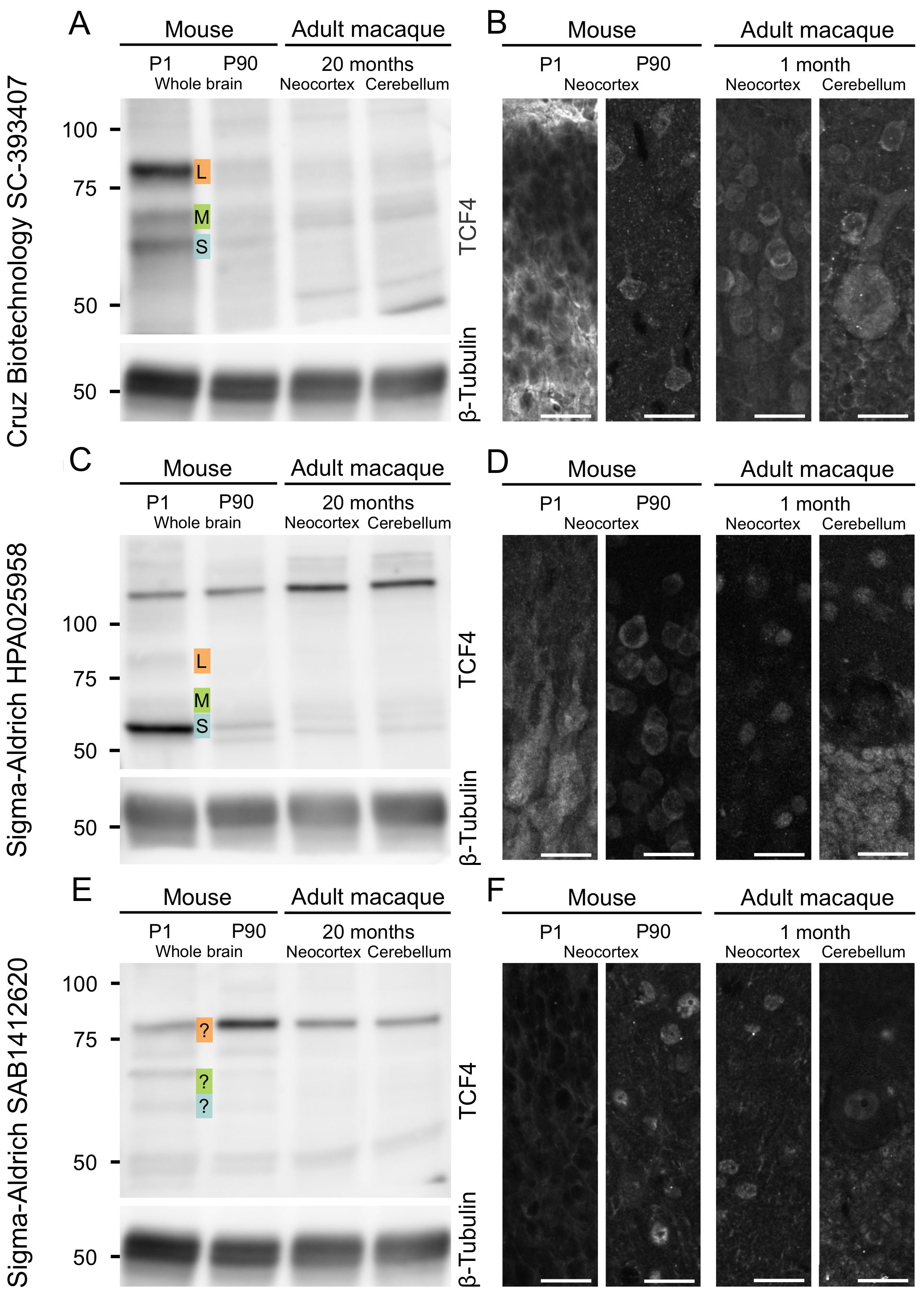

Supplement: Supplementary file 1 [file Image_1.JPEG]

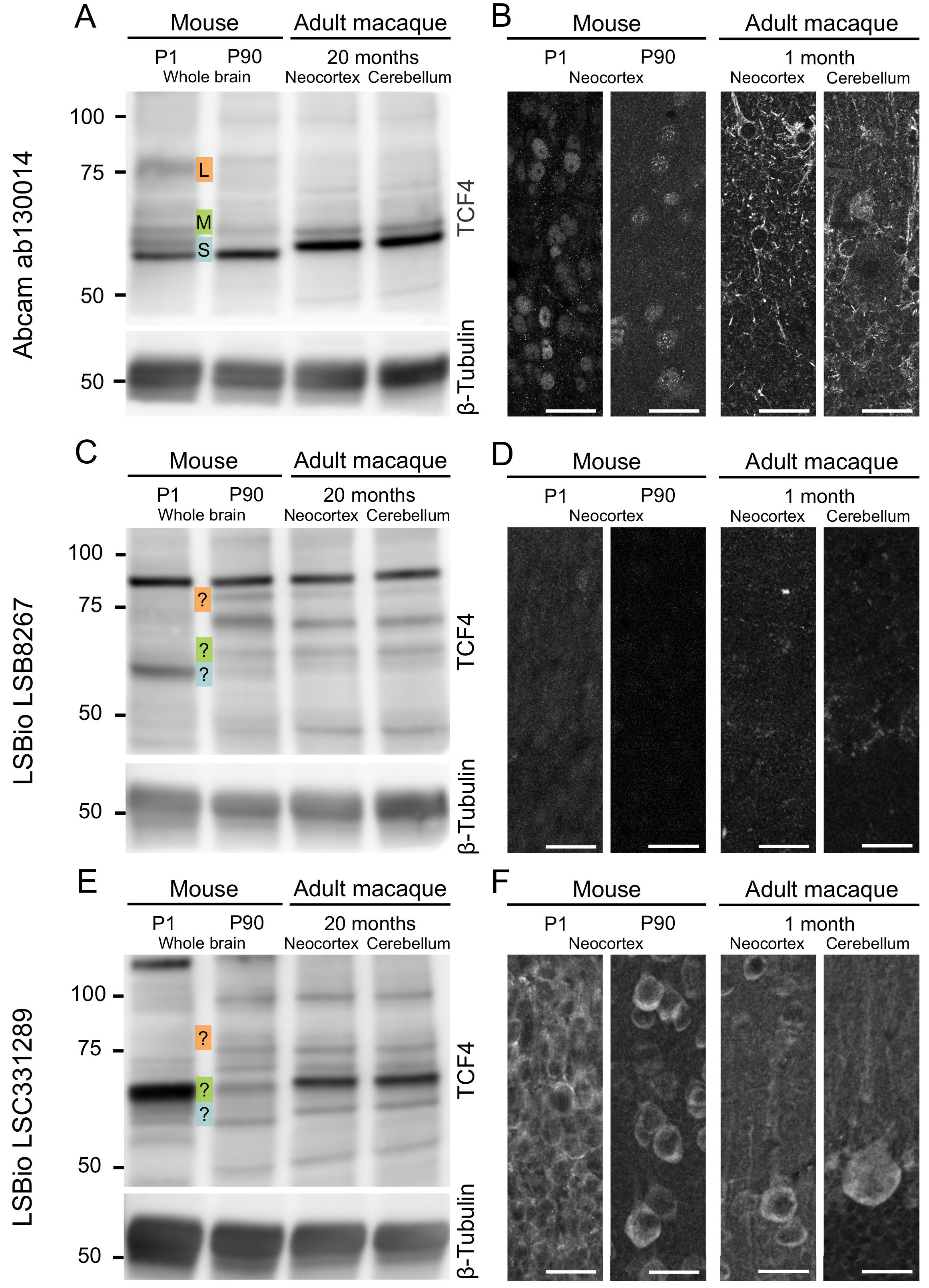

Supplement: Supplementary file 2 [file Image_2.JPEG]
